# Current Knowledge of the Potential Links between Inflammation and Prostate Cancer

**DOI:** 10.3390/ijms20153833

**Published:** 2019-08-06

**Authors:** Tommaso Cai, Raffaella Santi, Irene Tamanini, Ilaria Camilla Galli, Gianpaolo Perletti, Truls E. Bjerklund Johansen, Gabriella Nesi

**Affiliations:** 1 Department of Urology, Santa Chiara Regional Hospital, 38122 Trento, Italy; 2 Careggi University Hospital, 50134 Florence, Italy; 3 Department of Health Sciences, University of Florence, 50139 Florence, Italy; 4 Department of Biotechnology and Life Sciences, Università degli Studi dell’Insubria, 21100 Busto Arsizio, Italy; 5 Department of Urology, Oslo University Hospital, 0424 Oslo, Norway

**Keywords:** prostate cancer, inflammation, prostatitis, review, progression

## Abstract

Inflammation is inherent in prostatic diseases and it is now accepted that it may facilitate cellular proliferation in both benign and malignant conditions. The strong relationship between prostatic inflammation and pathogenesis of benign prostatic hyperplasia (BPH) is supported by epidemiologic, histopathologic and molecular evidence. Contrariwise, the role of inflammation in prostate carcinogenesis is still controversial, although current data indicate that the inflammatory microenvironment can regulate prostate cancer (PCa) growth and progression. Knowledge of the complex molecular landscape associated with chronic inflammation in the context of PCa may lead to the introduction and optimization of novel targeted therapies. In this perspective, evaluation of the inflammatory component in prostate specimens could be included in routine pathology reports.

## 1. Introduction

Prostate cancer (PCa), one of the most challenging malignancies in urologic oncology, is a very inhomogeneous disease. Advanced age, African descent, family history, and genetic mutations are well established risk factors but the mechanisms responsible for the development and progression of PCa have not yet been fully elucidated [1,2,3]. Several authors have focused their attention on the role of inflammation in the development of PCa based on the hypothesis that inflammatory injury could prompt carcinogenesis by causing cellular stress and repeated genomic damage [3,4,5]. This hypothesis has been extended from bench to bedside and numerous clinical studies have investigated the association between prostatitis and PCa without, however, reaching a universally accepted agreement [3]. While some authors have demonstrated an increased risk of PCa among men with previous prostatitis [6], others claim that prostatic inflammation may confer a protective effect and decrease the rate of subsequent PCa [7,8]. The latest systematic review and meta-analysis on the association between prostatitis and PCa has recently been compiled by Perletti and co-workers, who reported a significant increase in the odds of prostatic adenocarcinoma in patients with a history of clinical chronic prostatitis [3]. Their findings confirmed a previous meta-analysis by Jiang et al. establishing a correlation between clinical prostatitis and PCa [9].

This review attempts to discuss peer-reviewed data on the role of inflammation in PCa focusing on the following issues:

What is the prevalence of prostate inflammation in the general population and PCa patients?

What is the role of histologically demonstrated inflammation in PCa development and progression?

How can prostate inflammation be detected in everyday clinical practice?

## 2. Acquisition of Evidence

Search strategy and study selection:

Our research started with the latest meta-analysis performed by Perletti et al. in 2018 and was then extended to January 2019 to include all published papers assessing the role of asymptomatic and histologically confirmed prostatic inflammation in PCa genesis and progression [3]. Data extraction was carried out by consulting the most important international databases and trial registers, such as Medline, PreMedline, Embase, Cochrane Library, Web of Science, Latin American and Caribbean Health Sciences Literature (LILACS), Scopus, OpenGrey, the World Health Organization (WHO) International Clinical Trial Registry and clinicaltrials.gov. No systematic review or meta-analysis was accomplished.

## 3. Findings

### 3.1. What Is the Prevalence of Prostate Inflammation in the General Population and PCa Patients?

It is common knowledge that the prevalence of inflammation is very high within the adult prostate. The REduction by DUtasteride of PCa Events (REDUCE) trial demonstrated that 77.6% of all prostate biopsies exhibited inflammatory tissue and, of these, the majority (89%) had mild chronic inflammation [10]. Evidence of prostatic inflammatory tissue has been gathered not only from prostate biopsies, but also from radical prostatectomy specimens and tissue removed at transurethal resection for benign prostatic hyperplasia (BPH). Some authors outlined a prevalence of prostatic inflammation in the general population ranging from 33% to 100% [11,12]. Recently, Murtola and co-workers reported that 91.6% of PCa patients in the finasteride arm had at least one biopsy core with inflammation in benign areas, highlighting the high prevalence of inflammation associated with PCa [13]. This raises the following questions:

What are the cellular components of prostate inflammation?

What are the pathologic features of prostate inflammation?

What are the causes of prostate inflammation?

### 3.2. Cellular Components of Prostate Inflammation

Chronic inflammation is a common finding in malignant prostate tissue; the inflammatory infiltrate primarily consists of T lymphocytes, macrophages and, less frequently, plasma cells and eosinophils [12]. Miller et al. showed that the number of CD4 + CD25^high^ regulatory T cells (T_reg_) is markedly increased in both tumor tissue and peripheral blood of patients with early-stage PCa [14]. The available evidence confirms that the presence of infiltrating T_reg_, in PCa has significant implications in immunotherapy strategy planning. In addition, CD204+ macrophages and CD3+ T lymphocytes play a key role in tumor development, with the highest number of CD3+ cells being detected in PCa, and gradually decreasing in prostatic intraepithelial neoplasia (PIN) towards the lowest number in BPH [15].

Histologically, the prostate epithelium can be injured by numerous environmental factors such as inflammatory disorders. Procarcinogenic inflammatory processes may promote cell transformation, mediated by transcription factor NF-kB activation that leads to the upregulation of inflammatory cytokines such as TNF-α and IL-6 [16].

Currently, an evolving class of inflammasomes is deemed to be the master regulator of inflammation. Inflammasomes are a group of multimeric proteins consisting of NLR protein, an apoptosis-associated speck-like protein, which contains a carboxyterminal CARD (ASC), and procaspase-1 [17,18,19]. A wide variety of biologic effects associated with infection and inflammation are caused by the assembly of inflammasome complex, which activates caspase-1, and causes the secretion and maturation of proinflammatory cytokines IL-1β and IL-1 [17,20]. NLRP3 is one of the most studied inflammasomes [17]. In a model of chemical-induced carcinogenesis, Chow et al. demonstrated that in NLRP3-deficient mice display significantly reduced tumor burden compared to control wild-type mice. Indeed, NLRP3 deficiency induces NK cell infiltration and is associated with increased CCL5 and CXCL9 chemokine production [21] As a result of limited evidence, the contribution of inflammasomes to regulating prostate inflammation is still controversial. However, investigation into the role of inflammasomes may offer novel therapeutic options to target the complexities of inflammatory pathways [17].

Moreover, Sorrentino et al. demonstrated the role of IL30 in regulating prostate cancer stem-like cell behavior and metastatic potential, highlighting that IL-30 overproduction promoted tumor onset and development associated with increased proliferation, vascularization and myeloid cell recruitment [22]. IL-30 appears to have a pro-inflammatory potential since, just as IL-6, it is known to signal via IL-6Rα, by recruiting a gp130 protein receptor homodimer [23]. In this sense, inflammatory microenvironment may play a decisive role in promoting PCa development, progression and metastasis, although future studies are mandatory to understand this correlation more thoroughly.

### 3.3. Histopathologic Characteristics of Prostate Inflammation

In the pathology report, the term “prostatitis” describes the presence of inflammatory cells within the prostatic parenchyma, and cell types constituting the infiltrate (i.e., neutrophils, eosinophils, lymphocytes, plasma cells, macrophages) are given [24]. However, pathologists may tend to overlook prostatic inflammatory infiltrates unless particularly florid, since their clinical relevance remain largely undefined [24]. Moreover, while prostate biopsies with no interstitial inflammatory cells virtually do not exist, the point beyond which the prostate should be considered “inflamed” has not been fully established, nor any standardized reporting system for prostatitis routinely practiced.

Since the late-sixties, several clinico-pathologic studies have explored the presence of inflammation in prostate specimens within various clinical settings, adopting different criteria to describe pathologic features of inflammatory infiltrates [25,26,27,28,29,30,31,32,33,34].

Although the three primary locations of prostatic inflammation (i.e., glandular, periglandular and stromal) are generally recognized, no consensus has been reached regarding quantification of extent and grade of inflammation, neither has any consistent correlation emerged with clinical syndromes or symptoms. Various classification systems were published between 1991 and 2001 (see Supplementary Materials).

In 1991, Brawn and co-workers evaluated a series of clinically benign whole-mount prostate specimens obtained at autopsy, to establish whether any link existed between histologic findings and prostate specific antigen (PSA) concentrations. They categorized prostate inflammatory infiltrates as minimal, moderate or severe, but did not distinguish between acute and chronic inflammation. Severe inflammation was capable of elevating PSA levels (higher than 4 ng/mL) independent of other histologic findings [29].

In a large series of prostate needle biopsies obtained from men without PCa, Nadler et al. analyzed duration and type of infiltrating inflammatory cells, differentiating acute from chronic inflammation, which was graded on a 3-tiered scale: 0-none, 1-low grade and 2-high grade. Acute inflammation contributed the most to PSA elevation [31].

In 1997, Irani and co-workers, while attempting to correlate pre-biopsy serum and urinary PSA concentrations with morphologic characteristics of inflammation in benign prostatic biopsies, defined a 4-point scale for describing inflammatory infiltrates in terms of extension and aggressiveness [32]. This grading system did not include the types of inflammatory cells composing the infiltrate. The authors found no association between prostatic subclinical inflammation and high urinary PSA, nor any correlation between the bulk of prostatic inflammatory infiltrate and serum PSA concentration, except in the case of glandular epithelial disruption [32]. Subsequently, the same authors, having investigated the prognostic value of prostatic stromal inflammation in surgically treated localized PCa, proposed two categories of aggressiveness to describe prostate inflammation, low-grade and high-grade. Patients with high-grade inflammation surrounding malignant glands had significantly more postoperative biochemical recurrence than patients with low-grade inflammation [35].

Some investigators characterized prostatic inflammatory infiltrates by means of immunohistochemistry [36,37]. Anim et al. localized the different types of inflammatory cells in prostate specimens with acute/chronic active/chronic inactive prostatitis, to determine the sequence of events in the cellular response. They concluded that the reaction to prostatic injury/stimuli is initiated by macrophages, probably related to PSA, prostate specific acid phosphatase (PSAP) and other antigenic molecules that are normally present in prostatic secretion draining into the periglandular tissues. T-lymphocytes are then recruited to participate in the inflammatory response and accumulate around the damaged glands, whereas B-cell recruitment only occurs later [36].

Considering prostate histopathology in a series of patients affected by chronic prostatitis/chronic pelvic pain syndrome, True et al. analyzed prostatic inflammation for extent (on a 3-point scale), anatomic location (glandular, periglandular, stromal) and distribution (focal, multifocal, diffuse) of inflammatory cells. These authors, among the first to systematically characterize inflammation of the prostate, found that the degree of inflammation was minimal to absent in over 95% of cases and that severe inflammation was infrequent [33].

Conversely, according to Nickel et al., prostatic inflammation is an extremely common finding in BPH patients with no history or symptoms of prostatitis. The authors examined histologic sections from TURP specimens, using an immunostain for leukocyte-common antigen and a computerized image-analysis system, to assess the pattern of distribution and intensity of inflammatory infiltrates, following the model of Irani and co-workers. Neither total PSA nor PSA density showed a significant correlation with the extent, grade or location of inflammation [34].

**Figure 1:**
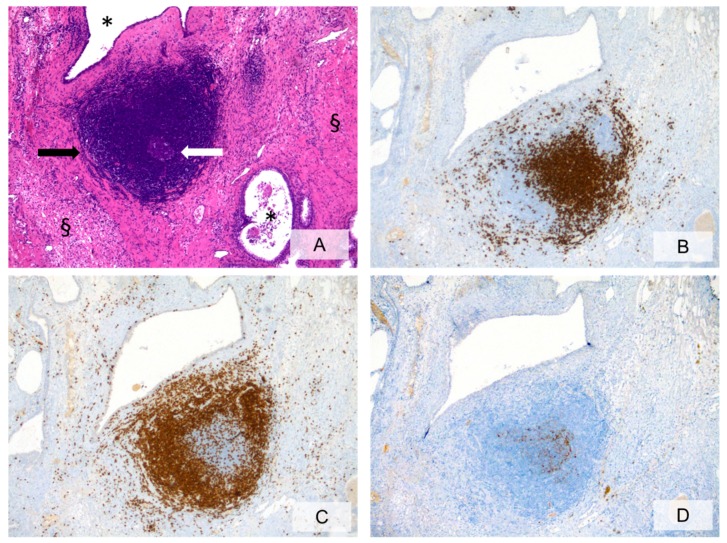
Prostatic stroma showing marked focal chronic inflammation with lymphoid follicle formation (**A**, H&E, ×10, black arrow = lymphoid follicle; white arrow= germinal center; * = prostatic glandular lumina; §= prostatic stroma;). Immunohistochemical staining for anti-CD20 (**B**, ×10) and anti-CD3 (**C**, ×10) highlights the B-cell and the T-cell compartments, respectively. Anti-CD21 staining (**D**, ×10) identifies the follicular dendritic cell meshworks in germinal centers.

**Figure 2:**
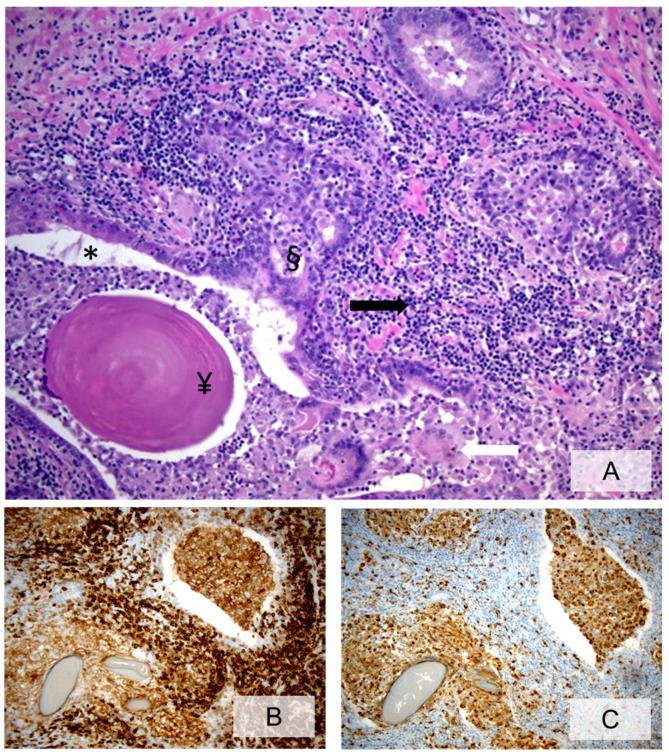
Heavy chronic inflammation with confluent areas surrounding prostatic glands, damaging glandular epithelium and involving glandular lumina. Inflammatory cells are represented by lymphocytes, histiocytes and scattered foreign-body-like giant cells (**A**, H&E, ×20, black arrow = confluent areas of chronic inflammatory infiltrate mainly consisting of lymphocytes; white arrow = foreign-body-like giant cells; * = prostatic glandular lumina; § = glandular epithelium; ¥ = corpora amylacea within glandular lumina). Immunohistochemical staining with anti-CD45 (**B**, ×20) and anti-CD68-PGM1 (**C**, ×20) highlights the lymphocyte and the histiocyte components, respectively.

**Figure 3:**
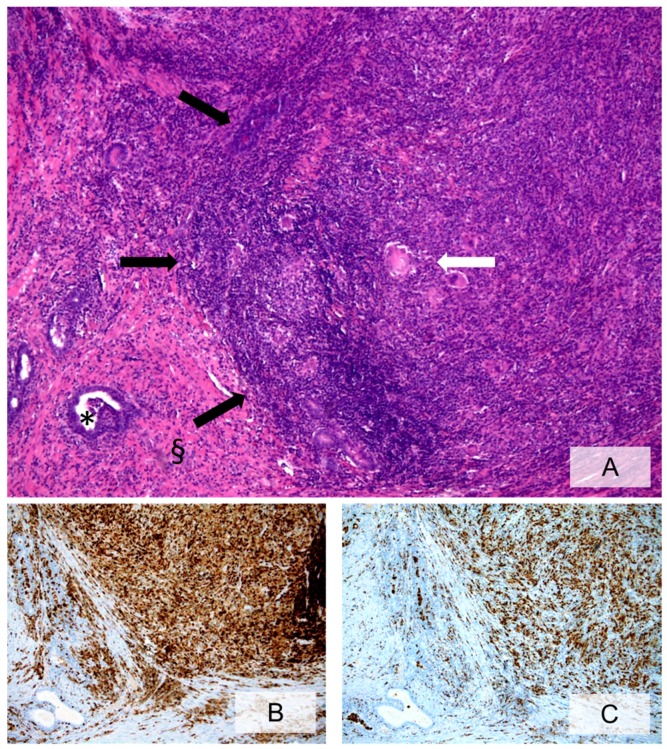
Granulomatous prostatic inflammation (**A**, H&E, ×10, black arrows = circumscribed granulomatous inflammatory infiltrate; occasional foreign-body-like giant cells within the granuloma, * = prostatic glandular lumina, § = prostatic stroma). Immunohistochemical staining with anti-CD45 (**B**, ×10) and anti-CD68-PGM1 (**C**, ×10) labels the lymphocyte and the histiocyte components, respectively.

In 2001, Nickel and co-workers, with the contribution of the participants of the North American Chronic Prostatitis Collaborative Research Network (CPCRN) and the International Prostatitis Collaborative Network (IPCN), proposed a consensus classification system of chronic prostatic inflammation, based on previously published critical research, describing inflammatory patterns in prostate specimens from patients with BPH [24]. Inflammatory infiltrates should be evaluated according to their location, extent and grade (Figure 1 and Figure 2). Of note, no consensus was reached on the frequency, relevance and classification of granulomatous inflammation; however, it was agreed to record its extent, location and severity (Figure 3). These authors reported several distinct, although often coexisting, patterns of chronic inflammation in the prostate, the most common being a lymphocytic infiltrate in the stroma adjacent to the prostatic acini with a less prominent intraepithelial component [27,33,34].

Periglandular inflammation is usually patchy and, the peripheral zone is more frequently affected, although other regions of the prostate can be involved [38,39]. Intensity can vary markedly, from scattered lymphocytes to dense lymphoid nodules or secondary follicles with germinal centers. In cases with copious periglandular inflammatory cells, the degree of epithelial infiltration may be higher and effects on the epithelial cells more noticeable, with the basal cells appearing more resistant to inflammatory damage [24]. Periglandular inflammatory infiltrates often consist of a mixed population of B and predominating T lymphocytes [40,41]. Macrophages also occur in significant numbers both in glands and in the periglandular stroma, while plasma cells are less common.

An accepted, standardized scaffold to describe prostatic inflammation will prove useful in better elucidating asymptomatic inflammatory prostatitis associated with BPH, PCa and infertility [42,43].

### 3.4. What Is the Role of Histologically Demonstrated Inflammation in PCa Development and Progression?

Preclinical studies provide a biologic rationale linking inflammation and the risk of PCa, but a direct relationship between inflammation and malignant transformation of human prostate is still under debate [44,45]. It has been hypothesized that immunologic response, induced by chronic prostatic inflammation, promotes tissue damage and subsequent repair, which may lead to enlargement and cancer vulnerability of the gland. After conducting a meta-analysis on 11 case-control studies published between 1971 and 1996, Dennis and co-workers showed an increased risk of PCa among men with a history of prostatitis [6]. Conversely, the first study to prospectively assess the linkage between prostatitis and PCa, performed by Sutcliffe et al. in 2006, failed to demonstrate any statistical correlation although a positive association was observed among younger men screened for PCa [46]. Moreover, in a cohort of 4526 men undergoing prostate biopsy, Karakiewicz et al. reported a protective effect of inflammation and decrease in the rate of subsequent PCa [7]. Along this line, the Finnish PCa screening trial [8] and the REDUCE study [47] showed PCa lower rates and decreased risk of PCa at 2-year repeat biopsy in patients with prostatic inflammation, respectively. On the other hand, a prospective study by Cheng et al. on 68,675 men revealed that a personal history of prostatitis and prolonged prostatitis symptoms can significantly increase the odds of PCa [48]. Compiling the results from all clinical trials published from May 1990 to July 2012, Jiang et al. confirmed that there is a positive relationship between clinical prostatitis and PCa [9]. The latest evidence on the role of prostatitis in the development and progression of PCa was gathered by Perletti et al. in 2017 [3]. This meta-analysis of 15 case-control studies showed a significant association between PCa and exposure to prostatitis, fostering investigation as to whether aggressive therapeutic management of chronic prostatitis syndromes could be instrumental in the prevention of cancer.

Recently, Aykan and co-workers revealed a correlation between male accessory gland infection (MAGI) and PCa in a case-control study [49]. They also observed that men with MAGI have a higher risk of clinically significant or high-grade PCa. In patients with a positive biopsy after an initial negative baseline biopsy, Kuang et al. demonstrated that acute and chronic inflammation is related to a lower prevalence of perineural invasion, although the mechanism for this association remains unclear [50].

### 3.5. What Are the Causes of Prostate Inflammation?

Several factors, including pathogens, diet, mechanical and chemical trauma, can trigger the clinical condition of prostatitis. The most common causes of prostate inflammation fall under infectious and noninfectious etiology.

#### 3.5.1. Infectious Etiology

Bacterial prostatitis is mainly secondary to Gram-negative uropathogens, although Gram-positive and sexually transmitted pathogens have also been identified as causative agents. Over the last few years, Cai et al. have reported a significant increase in the prevalence of Gram-positive strains in patients with chronic bacterial prostatitis, highlighting the role of atypical microorganisms in the onset of infections, probably due to the more frequent use of antibiotics [51] Chlamydia trachomatis has also been identified as an important pathogen in chronic prostatitis, with a serious impact on patient quality of life [52].

Bacteria produce protein toxins that could act as carcinogenetic stimuli [53]. Toxin-mediated carcinogenesis can occur in multiple ways, by affecting genomic instability, resistance to cell death and proliferative signaling [54].

#### 3.5.2. Noninfectious Etiology

Noninfectious causes can also lead to the development of prostate inflammation. Several authors have focused their attention on the role of diet, changes in serum testosterone and estrogen levels, autoimmunity, smoking and reflux of noxious chemicals in the urine. Metabolic syndrome and metabolic alterations also feature among noninfectious causes of prostate inflammation [41,42,43]. A high-fat diet can induce a significant increase in pro-inflammatory cytokines through activation of the Signal Transducer and Activator of Transcription (STAT)-3, and Nuclear Factor-kappa B (NF-kappaB) pathways. Both these pathways are involved in the transcriptional regulation of genes related to proliferation, survival, angiogenesis, invasion, and inflammation within the prostate [55,56]. In addition, several studies have shown that estrogens are implicated in the induction of inflammatory disease of the prostate in rats, suggesting that steroids modulate the immune response [57]. Table 1 lists circulating and in situ inflammatory factors detected in PCa patients [22,58,59,60,61,62,63,64].

### 3.6. How Can Prostate Inflammation Be Detected in Everyday Clinical Practice?

As mentioned above, Perletti et al. showed that a history of clinical chronic prostatitis can significantly increase the odds for PCa of any grade (OR = 1.83, 95% CI: 1.43 to 2.35), however, in prostatitis patients, who undergo more frequent urologic consultation and instrumentation, there is a strong probability of detecting indolent clinically irrelevant cancers [3]. The overlap of prostatitis and other urologic diseases, such as BPH, should also be taken into account. Indeed, reporting chronic prostatitis is subjective and symptoms related to prostatitis and BPH (likely prevalent in older subjects) may be difficult to distinguish. Since epidemiologic studies suggest an association between chronic inflammation and the risk of PCa, an aggressive diagnostic approach to chronic prostatitis as cancer-preventive strategy is crucial. Biopsy is the golden standard to detect the presence of inflammatory tissue in the prostate, however, it can only be performed in patients with suspected PCa [65]. In addition, there is a high prevalence of chronic prostatic inflammation in patients with lower urinary tract symptoms (LUTS) due to BPH, who are not candidates for prostate biopsy. Ficarra and co-workers proposed several parameters to be used in everyday clinical practice when suspecting prostatic inflammation [65]: laboratory parameters (e.g., cytokines), clinical parameters (e.g., prostatic calcifications, symptom severity, and response to therapy).

Laboratory parameters include interleukin-8 (IL-8), a proinflammatory cytokine produced by prostate epithelial cells. IL-8 promotes proliferation of prostate stromal cells in an autocrine/paracrine manner and is a potential link between chronic inflammation and prostate enlargement [63]. Monocyte chemotactic protein-1 (MCP-1) is another potential biomarker of prostatic inflammation. In prostatic secretion, MCP-1 levels have been shown to positively correlate with prostate volume and mRNA levels of the macrophage marker CD68 [64]. These are both promising biomarkers, although more robust evidence from prospective clinical studies is warranted before introducing predictive biomarkers into routine clinical practice to assist diagnosis and surveillance of chronic prostatic inflammation [64].

Prostatic calcifications are a common finding during transrectal prostate ultrasound in both healthy subjects and patients undergoing prostate biopsy. It is generally accepted that their incidence increases with age [66], they are somehow related to chronic prostatitis or chronic pelvic pain syndrome in young men [67,68], and appear to play an important role in LUTS [69]. Their etiopathogenesis is not yet fully understood. Some authors have demonstrated that microbial biofilms are critical in the genesis of prostate calcifications [68], while others report that prostatic calcifications, leading to obstruction of intraprostatic ducts, may result from prostatic fluid alteration and, in turn, induce chronic inflammation [70]. The inflammatory response is characterized by T-lymphocyte infiltration, activation and upregulation of proinflammatory cytokines, as well as increased expression of stromal and epithelial growth factors following abnormal prostate cell proliferation [63]. In this respect, calcifications are a clinical demonstration of prostatic inflammation.

A statistically significant correlation between chronic prostatic inflammation and total, irritative and obstructive symptoms has been shown by several authors [10]. In a cohort study of 82 patients undergoing transrectal ultrasound-guided needle prostate biopsy, Kwon and co-workers demonstrated that patients with high-grade chronic inflammation have significantly lower changes in the International Prostate Symptom Score (IPSS) than patients with low-grade inflammation over the 12-month follow-up period [71]. Moreover, patients with low-grade inflammation benefitted from continuous improvement of storage symptoms until 12 months after therapy with alpha-1 blockers and 5-alpha reductase inhibitors [5-ARIs]. No surgery was required in the low-grade group, while four patients (9.1%) in the high-grade group underwent transurethral resection [71]. These findings imply that the use of alpha blockers, with or without 5-ARIs, may not be sufficient to decrease symptom severity in patients with high-grade inflammation.

Several authors have found that serum IL-6 levels are elevated in patients with untreated metastatic or castration-resistant PCa and correlate negatively with tumor survival and response to chemotherapy [59]. Furthermore, a substantial body of evidence suggests that IL-6 plays a major role in the transition from hormone dependency to castration-resistant PCa, most notably through accessory activation of the androgen receptor [59]. Prostate inflammation, through IL-6 and IL-6-related pathways, thus seems to be crucial in metastatic or castration-resistant PCa, although further validation is needed on the use of anti-inflammatory drugs in patient management.

## 4. Clinical Remarks

**Figure 4:**
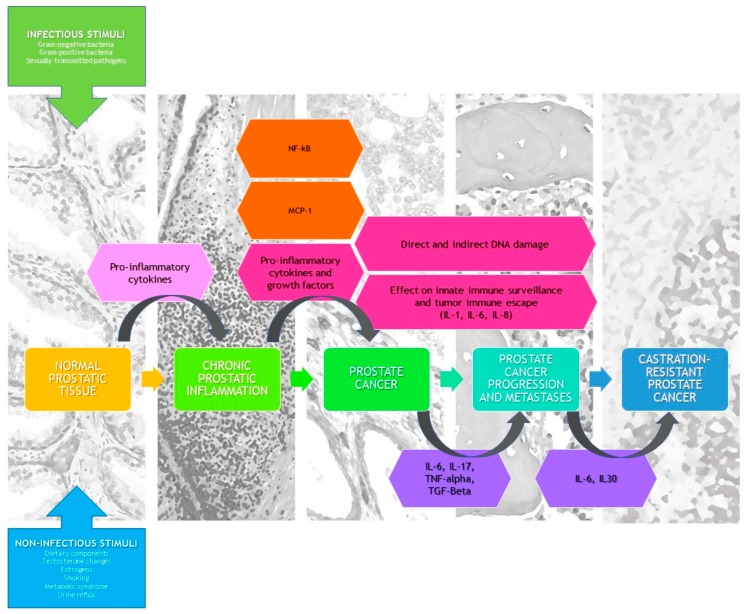
Impact of prostatic inflammation and inflammatory mediators on tumor initiation, growth and progression. NF-kB: Nuclear Factor-kappa B.

Chronic inflammation contributes to the onset and progression of human cancer via modifications in the tumor microenvironment, by remodeling the extracellular matrix and initiating epithelial mesenchymal transition [72]. The latest reviews and meta-analyses demonstrate a strong correlation between a history of clinical chronic prostatitis and PCa development in the general population [3]. Causes of prostate inflammation vary from bacteria triggering prostatitis and sexually-transmitted diseases, estrogen hormone imbalance, physical trauma, urine reflux to the prostate gland, and environmental factors such as diet [73,74]. Although prostate biopsy is the golden standard method for diagnosing prostate inflammation, several parameters, such as laboratory biomarkers (cytokines) and/or clinical parameters (prostatic calcifications, severity of symptoms, and response to therapy), could be used in everyday clinical practice when prostate inflammation is suspected [65]. Figure 4 shows the impact of prostatic inflammation and inflammatory mediators on tumor initiation, growth and progression.

One important question still remains unanswered: would preventive anti-inflammatory treatment efficiently avoid or delay the development of PCa in high-risk patients? Based on data from the EPIdemiology of Prostate Cancer (EPICAP) study, designed to investigate the role of environmental and genetic factors in PCa, Doat et al. recently reported a decreased risk of PCa in men treated with nonsteroidal anti-inflammatory drugs, particularly those with anti-COX-2 activity [75]. They also stated that the protective effect of nonsteroidal anti-inflammatory drugs seems to be more pronounced in aggressive PCa and in men with a personal history of prostatitis.

Further studies are needed to identify definitive clinical markers for prostate inflammation diagnosis and enable optimization of novel therapeutic modalities for the treatment of high-risk PCa patients.

## Figures and Tables

**Table 1:** Circulating and in situ inflammatory factors detected in PCa patients (PCa: Prostate Cancer).

	Description	Role	Reference
IL-1	Proinflammatory cytokine	Promotes cancer growth and progression	Voronov (2003) [58]
IL-6	Proinflammatory cytokine	Promotes PCa growth and progression; has a role in CRPC	Nguyen (2014) [59]
IL-8	Proinflammatory cytokine	Proliferation of prostate stromal cells; to regulates the expression of matrix metalloproteinases and PCa progression	Liu (2009) [63]
IL-17	Proinflammatory cytokine	Promotes PCa growth, angiogenesis and metastasis	Steiner (2003) [60]
IL-30	Proinflammatory cytokine	Promotes PCa progression and metastasis	Sorrentino (2018) [22]
MCP-1	Chemotactic cytokine	Promotes prostatic inflammation	Fujita (2010) [64]
TNF-α	Proinflammatory cytokine	Promotes PCa progression and metastasis	Michalaki (2004) [61]
TGF-β	Multifunctional cytokine	Promotes PCa progression and metastasis	Park (2003) [62]

Abbreviations: Interleukin (IL); Monocyte Chemoattractant Protein (MCP); Tumor Necrosis Factor (TNF); Transforming Growth Factor (TGF); Castration Resistant Prostate Cancer (CRPC).

## Supplementary Materials

Supplementary materials can be found at https://www.mdpi.com/1422-0067/20/15/3833/s1.
