# Supplementary material for: Current Knowledge of the Potential Links between Inflammation and Prostate Cancer
Source: Int J Mol Sci. 2019 Aug 6;20(15):3833. doi: 10.3390/ijms20153833 (PMC6696519; doi:10.3390/ijms20153833)
Supplement: Supplementary file 1 [file ijms-20-03833-s001.pdf]

**Brawn, 1991**	Minimal to no inflammation		Occasional inflammatory cells, usually lymphocytes, primarily in stroma with minimal involvement of prostatic glands	
	Moderate		Involving larger portion of prostate; acute, chronic or both, occupying less than one low-power (5-mm) microscopic field	
	Severe		Acute, chronic, or both, filling at least one low-power microscopic field	
**Nadler, 1995**	Type of inflammation	Acute prostatitis	Polymorphonuclear leukocytes in glandular or ductal lumina, their epithelium and/or adjacent stroma	
		Chronic prostatitis	Mononuclear cell infiltrate (lymphocytes, monocytes and plasma cells) in prostatic stroma, with inflammatory cells occasionally invading glandular epithelium	
	Grade of inflammation	0 (Any)	Not otherwise specified	
		1 (Low)	Not otherwise specified	
		2 (High)	Not otherwise specified	
**Irani, 1997**	Extent	0	No inflammatory cells	
		1	Scattered inflammatory cell infiltrate	
		2	Non-confluent lymphoid nodules	
		3	Large inflammatory areas with confluence of infiltrate	
	Aggressiveness	0	No contact between inflammatory cells and glandular epithelium	
		1	Contact between inflammatory cells and glandular epithelium	
		2	Glandular epithelium disruption in <25% of examined material	
		3	Glandular epithelium disruption in >25% of examined material	
**Anim, 1998**	Type of inflammation	Acute prostatitis	Foci of active glandular destruction with epithelial necrosis, presence of neutrophils and cellular debris in glandular lumen. Large numbers of neutrophils, macrophages and lymphocytes in stroma surrounding damaged glands. Small foci of chronic prostatitis may occur	
		Chronic inactive prostatitis	Damaged gland regeneration. Periglandular fibrosis common. Inflammatory infiltrate less florid than in chronic active prostatitis with lymphoid follicles occupying sites of completely destroyed glands	
		Chronic active prostatitis	Chronic inflammatory foci, areas of glandular destruction with periglandular infiltrates of lymphocytes and scattered macrophages. Partial necrosis or regeneration of glandular epithelium with inflammatory cells in epithelial layers. Secretion or corpora amylaceae usually seen in glandular lumina	
	Distribution of cells	Periglandular		
		Scattered		
		Follicle centre		
		Perifollicular		
		Lining cells		
		Lumen cells		
**True, 1999**	Density	Mild	<10 inflammatory cells per 1-mm microscopic field	
		Moderate	10-200 inflammatory cells per 1-mm. microscopic field	
		Severe	>200 inflammatory cells per 1-mm. microscopic field	
	Location	Glandular	In the lumina of or epithelium lining the glands	
		Periglandular	Within 50µm. of gland (s)	Focal (only 1 gland)
				Multifocal (multiple glands)

		Stromal:	In prostatic stroma, not centered around glands)	Focal (restricted to a 1-mm microscopic field)
				Multifocal
				Diffuse
**Irani, 1999**	High-grade	Inflammatory cell nodules with confluence of infiltrate and/or clear glandular epithelium disruption associated with interstitial inflammatory infiltrate		
	Low-grade	Absence of any of the criteria for high-grade inflammation		
**Nickel, 1999**	Pattern	Glandular		
		Periglandular		
		Stromal		
	Grade	1		
		2		
		3		
**Nickel, 2001**	Anatomic location (Histologic pattern)	Glandular	Inflammatory infiltrates in duct/gland epithelium and/or lumina	
		Periglandular	Inflammatory infiltrates in stroma, around prostatic ducts/glands, within 50 µm distance	
		Stromal	Inflammatory cells in prostatic stroma, not centered around prostatic glands/ducts and ≥50 µm distant	
	Extent (Tissue area involved by inflammatory cell infiltrates)	Focal	<10%	
		Multifocal	10-50%	
		Diffuse	>50%	
	Grade (Morphologic description – typical inflammatory cell density, cells/mm^2)	1/mild	Individual inflammatory cells separated by distinct intervening spaces (<100)	
		2/moderate	Confluent sheets of inflammatory cells without tissue destruction nor lymphoid nodule/follicle formation (100±500)	
		3/severe	Confluent sheets of inflammatory cells with tissue destruction or nodule/follicle formation (>500)	

Table 1. Histologic classification systems for prostate inflammation according to the literature.
